# *Bacillus cereus*-derived α-amylase disrupts biofilm formation and quorum sensing in multidrug-resistant *Klebsiella pneumoniae*

**DOI:** 10.1186/s12866-025-04301-z

**Published:** 2025-08-29

**Authors:** Abd-El-Rahman A. Mustafa, Amal M. Abo-Kamer, Lamiaa A. Al-Madboly

**Affiliations:** Department of Microbiology and Immunology, Faculty of Pharmacy, Tanta University, Tanta, Egypt

**Keywords:** Biofilm, *K. pneumoniae*, *Bacillus cereus*, CLSM, QRT-PCR, MBIC, MBEC, *RpoB*, *FimH*, *PapC* genes, MIC, MBC

## Abstract

**Background and objectives:**

*Klebsiella pneumoniae* is a multidrug-resistant pathogen implicated in severe community- and hospital-acquired infections such as bacteremia, urinary tract infections, sepsis, and pneumonia. Biofilm formation, driven by extracellular polymeric substances (EPS), enhances its persistence and resistance to antibiotics. This study evaluated the anti-biofilm, antibacterial, and quorum-quenching activities of a novel α-amylase *B. cereus*-derived α-amylase against clinical isolates of *K. pneumoniae*.

**Methods:**

The anti-biofilm activity of the enzyme was assessed via minimum biofilm inhibitory concentration (MBIC) and minimum biofilm eradication concentration (MBEC) assays. Biofilm architecture and viability were analyzed using confocal laser scanning microscopy (CLSM) with live/dead staining. Antibacterial efficacy was determined through minimum inhibitory concentration (MIC) and minimum bactericidal concentration (MBC) assays. Quorum-quenching effects were evaluated using qRT-PCR to assess the expression of biofilm-associated genes (*fimH* and *mrkD*), normalized to *rpoB*.

**Results:**

*B. cereus*-derived α-amylase exhibited MBIC and MBEC values of 64 µg/ml and 128 µg/ml, respectively; MIC and MBC ranged from 32 to 128 µg/ml. The *B. cereus*-derived α-amylase enzyme inhibited biofilm formation by approximately 79% ± 0.69, compared to 58% ± 2.06 by commercial α-amylase. Biofilm thickness was reduced from 179 μm to ~ 39 μm and ~ 73 μm following treatment with B. cereus-derived and commercial α-amylase, respectively. Live/dead ratios shifted significantly from 97/3% (untreated) to ~ 54/46% and 73/27% after treatment with *B. cereus*-derived and commercial α-amylase enzymes, respectively. Quorum-sensing gene expression was markedly downregulated following treatment with ½ MIC of *B. cereus*-derived α-amylase: *fimH* to 0.247 ± 0.045 (75.3% reduction) and *mrkD* to 0.187 ± 0.035 (81.3% reduction).

**Conclusion:**

*B. cereus*-derived α-amylase exhibited potent anti-biofilm, antibacterial, and quorum-quenching activities against *K. pneumoniae* clinical isolates. These findings highlight its potential as a novel therapeutic agent for managing biofilm-associated infections, either alone or as an adjunct to conventional treatments.

## Introduction

*Klebsiella pneumoniae*, a nonmotile, Gram-negative bacillus, is a major cause of urinary tract infections, pneumonia, bacteremia, liver abscesses, and wound infections. It poses a significant threat to hospitalized and immunocompromised patients due to its extensive virulence factors and antimicrobial resistance (AMR). Globally, *K. pneumoniae* contributes to 4–15% of septicemia cases, 2–4% of wound infections, and 3–30% of neonatal septicemia. Its pathogenicity is mediated by factors such as capsular polysaccharides, lipopolysaccharides (LPS), siderophores, hypermucoviscosity, and adhesion molecules, which facilitate colonization and infection [1].

A key determinant of *K. pneumoniae* virulence is its ability to form biofilms, which protect the bacteria from host immune responses and antimicrobial agents. Biofilms are complex microbial communities adhered to biotic or abiotic surfaces and embedded in a matrix of extracellular polymeric substances (EPS) comprising polysaccharides, proteins, lipids, and extracellular DNA (eDNA). This biofilm matrix not only impedes antibiotic penetration but also fosters the persistence of bacterial populations by promoting genetic exchange and the formation of persister cells. The development of *K. pneumoniae* biofilms follows distinct stages—adhesion, microcolony formation, maturation, and dispersion—each driven by bacterial motility, adhesion, and metabolic activity [2].

Fimbriae play a critical role in biofilm development and pathogenesis. Type 1 and type 3 fimbriae, encoded by the *fim* and *mrk* gene clusters, respectively, mediate adherence to both host tissues and abiotic surfaces such as catheters. The adhesion protein MrkD, located at the apex of type 3 fimbriae, enhances colonization of respiratory and urinary epithelial surfaces. Similarly, type 1 fimbriae, with their mannose-binding adhesion protein FimH, are integral to biofilm formation, particularly on abiotic surfaces [3].

In addition to biofilm formation, quorum sensing (QS) which is a crucial mechanism regulates *K. pneumoniae* pathogenicity by coordinating gene expression in response to population density. The impact of quorum-sensing signaling molecules (QSMs) on the expression of various genes, involved in biofilm formation can be determined by quantifying the extracellular concentrations of secreted autoinducer molecules. For example, *N*-acylhomoserine lactones (AHLs) mediate QS in Gram-negative bacteria, processes like biofilm formation and virulence factor production. Enzymatic degradation of AHLs, a strategy known as quorum quenching (QQ), offers a promising approach to disrupt QS-regulated processes and mitigate biofilm-associated infections [4].

Advanced imaging techniques such as confocal laser scanning microscopy (CLSM) have facilitated the study of biofilm architecture, development, and viability. By enabling high-resolution, three-dimensional visualization, CLSM has become indispensable in biofilm research [5, 6]. This study leverages CLSM to evaluate the anti-biofilm activity of α-amylase against *K. pneumoniae* biofilms, with a focus on its effects on QS-regulated genes.

## Materials and methods

### Test bacterial isolates

A total of 25 *K. pneumoniae* isolates (respiratory pathogens) were obtained from the Department of Microbiology and Immunology, Faculty of Pharmacy, Tanta University, Tanta, Egypt, and identified using the Vitek 2 compact system. They were stored in an ultrafreezer at −80 °C to be used in the analysis of the antibacterial activities of the α-amylase enzyme. A standard *K. pneumoniae* (ATCC 700603) was used as a positive biofilm-forming strain in the current study. It was purchased from the Department of Molecular Biology, Agricultural Research Center in Giza, Egypt.

### Preparation of α-amylase enzyme

α-amylase was previously extracted and purified from *Bacillus cereus* soil bacteria to be used for qualitative and quantitative assessments of *K. pneumonia* biofilm [7].

Commercial α-amylase prepared from *Bacillus amyloliquefaciens* strain was bought from Sigma Aldrich (Germany) to be utilised as an authentic sample for purposes of comparing against the activities of the *B. cereus*-derived α-amylase enzyme. The later was formerly extracted and purified through precipitation by organic solvent (chilled ethanol) [8]. Briefly, the culture broth was centrifuged at 10,000 rpm for 15 min at 4 °C, and the clear supernatant was collected and concentrated using a rotary evaporator. Undesirable proteins were removed by heating at 45 °C for 30 min, followed by cooling. The enzyme-containing supernatant was then subjected to ammonium sulphate precipitation at varying saturation levels (20–80%) in phosphate buffer (pH 6.5). After stirring in an ice-salt bath and centrifugation, the precipitate was dissolved in buffer. The most active fraction was dialyzed overnight using Millipore membranes to eliminate salts, filtered, and tested for enzyme activity (DNS method) and protein content (Lowry method).

In another purification step, the crude enzyme was treated with chilled ethanol at different concentrations (0–100%) while stirring at 4 °C. The precipitated proteins were collected by centrifugation and analyzed for amylase activity and protein content [8].

The *B. cereus*-derived α-amylase enzyme’s molecular weight was determined using SDS-PAGE with 7.5% stacking and 10% resolving gels. Samples were prepared with buffer, boiled, and electrophoresed at 120 V. The gel was stained with Coomassie Blue, and the molecular weight was estimated by comparing migration distances with standard protein markers [8].

### Biochemical identification of test bacterial isolates

According to the manufacturer’s protocol, a densitometer (BioMérieux, France) was employed to standardize the bacterial suspension to a turbidity equivalent to the 0.5 McFarland standard. Individual colonies were selected from a nutrient agar slant and suspended in 2.5 mL of sterile 0.45% aqueous saline solution (pH 7.0). The identification of Gram-negative (GN) bacteria was performed by inoculating the NF-64 card/GN cassette and entering the data into the VITEK^®^2 7.01 software (BioMérieux, France) within 30 min of inoculation. A reference strain of Escherichia coli (ATCC 8729) was included to ensure normalization of the results. The VITEK^®^2 system operates through an array of 47 biochemical tests, assessing resistance, enzymatic activity, and carbon source utilization, among other parameters. The identification process, which utilizes growth-based automated technology, required approximately eight hours for completion [9].

### Evaluation of MIC and MBC of α-amylase enzyme against clinical isolates of *K. pneumoniae*

The densitometer (BioMérieux, France) was utilized to adjust the bacterial suspension to a 0.5 McFarland turbidity standard following the manufacturer’s instructions. Each time, a single colony was selected from the nutrient agar slant and suspended in 2.5 mL of 0.45% sterile aqueous saline (pH 7.0). To identify Gram-negative bacteria, the data was entered into the VITEK^®^2 7.01 software (Biomerieux, France) within 30 min of inoculation into the NF-64 card/GN cassette. The test included 25 clinical isolates of *K. pneumoniae* using a reference standard sample for normalization of the results. The software relies on 47 biochemical tests measuring resistance, enzymatic activities, carbon source utilization, and more. It took approximately eight hours for the identification findings to become available. The VITEK 2 is an automated microbiology system utilizing growth-based technology for bacterial identification. For MIC and MBC determination, the MIC and MBC levels of α-amylase previously extracted from *B. cereus* were assessed using the broth microdilution method (EUCAST, 2024) [10]. For normalization and comparison of results, a commercial α-amylase enzyme from *B. amyloliqufaciens* (Sigma Aldrich, Germany) was selected as a reference standard. The reference standard biofilm forming *K. pneumoniae* (ATCC 700603) was utilized for result comparison. Each of the 25 bacterial isolates of *K. pneumoniae* was inoculated into sterile normal MHB media at a concentration of 0.5 McFarland. Then, wells of a microtiter plate were filled with 100 µl of MHB media, and *B. cereus*-derived α-amylase was added (100 µl) to each well to serially dilute it from 512 to 32 µg/ml. Subsequently, bacterial suspension of each test isolate was added to test wells followed by incubation at 37 °C for 24 h. The positive control are wells in which bacterial growth is observed, and the negative control are wells containing only the culture medium. The MIC was estimated as the lowest concentration of enzyme that inhibits visible bacterial growth. Cultures with concentrations equal to or above the MIC were transferred onto tryptone soya agar medium and re-incubated for 24 h to measure MBC levels. Plates with no visible colonies indicate that this concentration kills 99.98% of the bacteria and was defined as the MBC. The test was also performed using the commercial α-amylase as a reference. If the same result was obtained in triplicate repeated experiments, the values were set as MIC and MBC values [12].

### Screening for biofilm-forming *K. pneumoniae* isolates

This qualitative assessment for biofilm-forming *K. pneumoniae* isolates was carried out using two methods: the tube and Congo red agar methods [8].

### Tube method

Polystyrene tubes containing 10 ml of trypticase soy broth (Oxoid, UK) supplemented with 1% glucose were inoculated with a loopful of a single colony of overnight-cultured test bacteria. After incubation at 37 °C for 24 h, the tubes were emptied, gently rinsed with phosphate-buffered saline (PBS, pH 7.4), and left until complete dryness. Next, all test tubes were stained with 0.1% crystal violet (Oxoid, UK) for 15 min then the excess stain was eliminated by rinsing the tubes with deionized water. The tubes were then dried in an inverted orientation and examined for biofilm formation. A visible film covering the bottom and wall of the tube signified a positive outcome for the ability to form a biofilm [8].

### Congo red agar (CRA) media

Congo red agar (CRA) comprises brain heart infusion broth (Oxoid, UK) at 37 g/l, sucrose at 50 g/l, agar number 1 at 10 g/l, and Congo red at 0.8 g/l. A concentrated aqueous solution of Congo red stain was initially prepared and subsequently autoclaved at 121 °C for 15 min. Thereafter, the autoclaved BHI agar containing sucrose was incorporated into the solution at 55 °C. The prepared CRA plates were infected with isolated bacterial pathogens and incubated aerobically at 37 °C for 24 h. The appearance of black dry crystalline colonies on the CRA plates signified biofilm production, whereas pink or red colonies showed the absence of biofilm generation [8].

### Antibiofilm effect of *B. cereus*-derived α-amylase against *Klebsiella pneumoniae*

#### Biofilm formation and Inhibition assay

This experiment aimed to quantitatively assess the biofilm-forming capacity of *K. pneumoniae* isolates using a microtiter plate-based spectrophotometric assay and to evaluate the biofilm inhibitory effect of *B. cereus*-derived α-amylase. Overnight cultures of the test isolates were used to inoculate tryptic soy broth (TSB; Oxoid, UK), adjusted to pH 7 and standardized to 0.5 McFarland (1.5 × 10⁸ CFU/mL). The inocula were dispensed into sterile 96-well microtiter plates and incubated at 37 °C for 24 h to allow biofilm formation. After incubation, planktonic cells were removed by aspiration, and the wells were gently washed three times with sterile PBS (pH 7.4), air-dried for 15 min, and fixed with absolute ethanol for 30 min. The biofilms were then stained with 0.1% (w/v) crystal violet for 15 min, rinsed with distilled water, and dried. Bound dye was solubilized with 33% (v/v) glacial acetic acid, and absorbance was measured at 595 nm (SHIMADZU UV-2450, Japan). Untreated biofilms and sterile medium served as positive and negative controls, respectively [7]. To evaluate the antibiofilm activity of *B. cereus*-derived or commercial α-amylase, an appropriate volume of the test enzyme was added to TSB containing bacterial suspensions in each well to achieve a final concentration of ½ MIC. The plates were then incubated at 37 °C for 24 h. Controls consisted of bacterial suspensions without the enzyme (positive control) and medium alone (negative control). A commercial α-amylase served as a reference. All experiments were conducted in triplicate, and biofilm inhibition (%) was calculated as previously described [7].

$$\begin{aligned}&\text{Percent}{\text{ }}\text{of}{\text{ }}\text{biofilm}{\text{ }}\text{inhibition}{\text{ }} \\&= {\text{ }}(\text{Optical}{\text{ }}\text{density}\,\text{of}{\text{ }}\text{control}{\text{ }}\text{sample}{\text{ }}{\text{ }}\\&\quad - {\text{ }}\text{Optical}\,\text{density}{\text{ }}\text{of}{\text{ }}\text{test}{\text{ }}\text{sample}{\text{ }}\\&\quad/\text{Optical}{\text{ }}\text{density}\,\text{of}{\text{ }}\text{control}{\text{ }}\text{sample}{\text{ }}\times100\end{aligned}$$

#### Quantitative determination of MBIC and MBEC values of α-amylase against *K. pneumoniae* using the microtiter plate assay

This study evaluated the antibiofilm efficacy of *B. cereus*-derived α-amylase from *B. cereus* against *K. pneumoniae* biofilms, using a commercial *B. amyloliquefaciens* α-amylase (Sigma-Aldrich, Germany) as reference. Both minimum biofilm inhibitory (MBIC) and eradication (MBEC) concentrations were determined through standardized assays.

To determine MBIC, 50 µL aliquots of serially diluted α-amylase (512–16 µg/mL) were added to the wells in addition to the test bacterial suspension (0.5 McFarland standard, 1 × 10⁸ CFU/mL in TSB), followed by incubation at 37 °C for 48 h [7].

Pre-established biofilms were developed by incubating *K. pneumoniae* isolates (0.5 McFarland standard, 1 × 10⁸ CFU/mL in TSB) in 96-well polystyrene plates at 37 °C for 5 days, with media replenishment every 48 h. For MBEC determination, the spent media were carefully aspirated, and pre-formed biofilms were treated with fresh media containing enzyme solutions (final concentration range: 512–16 µg/mL), followed by incubation at 37 °C for 48 h [7].

Post-treatment, biofilms (in both cases) were washed with PBS, fixed with ethanol, stained with 0.1% crystal violet, and solubilized with 33% glacial acetic acid. Absorbance was measured at 610 nm (PowerWaveX microplate reader, Bio-Tek, USA). Sterile medium and untreated biofilms served as negative and positive controls, respectively.MBIC was defined as the lowest enzyme concentration inhibiting ≥ 80% biofilm formation relative to positive controls, while MBEC represented the minimal concentration achieving ≥ 99% eradication, confirmed by both visual inspection and negative subcultures on TSA plates (Oxoid, UK). All experiments were performed in triplicate with mean values reported [7].

### Analysis of biofilm thickness and live/dead cell ratios using CLSM and Fiji software

#### Biofilm formation and staining procedure

*Klebsiella pneumoniae* biofilms were cultivated in eight-well chamber slides with or without ½ MIC of the test enzyme treatment, following established protocols [10]. Following incubation, both control (untreated) and experimental wells (treated with either commercial or *B. cereus*-derived α-amylase) were subjected to viability staining.

A freshly prepared 1:1 mixture of acridine orange (AO) and propidium iodide (PI) (5 µl of 0.01 mg/ml each in 0.9% NaCl) was applied to distinguish viable from nonviable cells. The fluorescent dyes were used according to manufacturer’s specifications, with excitation at 488 nm and emission detection at 630 nm. This dual-staining approach enabled clear differentiation between metabolically active cells (green fluorescence) and membrane-compromised cells (red fluorescence).

Slides were incubated at room temperature (25 °C) in the dark for 10 min, then examined under a laser scanning confocal microscope (Leica DMi8, Germany). The experiment was repeated three times, and mean values were calculated. AO is a cell-permeable dye that fluoresces green when bound to double-stranded DNA, while PI penetrates only cells with compromised membranes, fluorescing red. Hence, viable cells appear green, and dead cells red [11].

#### Calculation of biofilm thickness Inhibition

Biofilm thickness was measured using CLSM, and inhibition was calculated using the following formula:

$$\begin{aligned}&\text{Percent}{\text{ }}\text{of}{\text{ }}\text{thickness}{\text{ }}\text{inhibition}{\text{ }} \\&= {\text{ }}\text{thickness}{\text{ }}\text{of}{\text{ }}\text{control}{\text{ }}\text{sample}{\text{ }}\left( {\mu m} \right){\text{ }}\\&\quad - {\text{ }}\text{thickness}{\text{ }}\text{of}{\text{ }}\text{test}{\text{ }}\text{sample}{\text{ }}\left( {\mu m} \right)\\&\quad/\text{thickness}{\text{ }}\text{of}{\text{ }}\text{control}{\text{ }}\text{sample}{\text{ }}\left( {\mu m} \right)\times100\end{aligned}$$

#### CLSM-Based quantification of live/dead cells using Fiji software

Quantification of live and dead cells within *Klebsiella pneumoniae* biofilms was performed using confocal laser scanning microscopy (CLSM) images analyzed with Fiji software (ImageJ, NIH, USA). Confocal images in “.czi” format were imported into the Fiji interface, and fluorescence channels were separated by applying the “Split Channels” function under the “Image → Color” menu. Thresholding was conducted on the brightest image frames by selecting “Image → Adjust → Threshold,” followed by applying default settings to convert images to binary format. Watershed segmentation was then applied (“Process → Binary → Watershed”) to differentiate overlapping cells. Particle analysis was carried out using the “Analyze → Analyze Particles” tool, with the size range set to 0–infinity and the “Add to Manager” option enabled. The “ROI Manager” window was accessed to select all identified regions of interest (ROIs). Color segmentation was applied via a pre-installed plugin by selecting dark live, bright live, and dead regions using the multi-point tool, after which the “Run” command was executed. A summary table showing the area percentage of each segmented region was generated. The results were exported to Microsoft Excel for further analysis. Live cell percentages were calculated by dividing the number of green-stained particles by the total number of fluorescent particles (green + red), and multiplying by 100%. Dead cell percentages were similarly calculated. Each image was analyzed in four technical replicates across three independent biological experiments, and values were reported as means.

Live/Dead Cell Ratio Calculations are done via the following equations:

$$\text{Total}\, \text{Cell}\, \text{Number} = \text{Live}\, \text{Cells} + \text{Dead}\, \text{Cells}.$$

$$\begin{aligned}&\text{Percentage}\, \text{of}\, \text{Live}\, \text{Cells} = (\text{Live}\, \text{Cells}/\text{Total}\, \text{Cell}\, \text{Number})\\&\quad\quad\quad\quad\quad\quad\quad\quad\quad\quad\quad \times 100.\end{aligned}$$

$$\begin{aligned}&\text{Percentage}\, \text{of}\, \text{Dead}\, \text{Cells} = (\text{Dead}\, \text{Cells}/\text{Total}\, \text{Cell}\, \text{Number}) \\&\quad\quad\quad\quad\quad\quad\quad\quad\quad\quad\quad\quad\times100.\end{aligned}$$

Viable cell percentages were determined by dividing the number of green-stained particles by the total number of particles (green + red), then multiplying by 100%. Each image was analyzed in four technical replicates per biological sample [12]– [13].

### Quantitative assessment of Quorum-Quenching activity of α-amylase on biofilm-related genes in *Klebsiella pneumoniae* using qRT-PCR

#### RNA extraction, cDNA synthesis, and qRT-PCR protocol

This assay was conducted to evaluate the effect of the *B. cereus*-derived α-amylase from *Bacillus cereus* on the expression of biofilm-associated genes (*fimH* and *mrkD*) in *K. pneumoniae* following treatment with ½ MIC (32 µg/ml) of the enzyme. Cultures without enzyme treatment served as positive controls. The *rpoB* gene was used as a housekeeping reference for normalization.

A colony of a clinically isolated biofilm-forming *K. pneumoniae* strain was cultured overnight in Luria Bertani (LB) broth, then subcultured and adjusted to ~ 2 × 10⁶ CFU/ml in 1.5 ml volumes. After 6 h of incubation with or without the α-amylase at 37 °C, total RNA was extracted using an RNA extraction kit (Applied Biosystems, USA). RNA samples were treated with RNase-free DNase for 10 min and quantified using a NanoDrop spectrophotometer (Thermo Scientific, USA) by measuring the A260/A280 ratio. RNA samples were stored at − 70 °C for further use.

Complementary DNA (cDNA) synthesis was performed using a commercial reverse transcription kit following the manufacturer’s instructions, and cDNA was stored at − 70 °C for subsequent qRT-PCR analysis. Primers specific to *fimH* and *mrkD* (Table 1) were obtained from Macrogen Labs (Korea). qRT-PCR reactions were conducted using SYBR Green/ROX qPCR Master Mix (Applied Biotech, USA) in 96-well plates. Table 2 showed that each 25 µl reaction contained 3 µl of cDNA, 12.5 µl of master mix, 1 µl each of forward and reverse primers, 1.2 µl of template buffer, and 7.3 µl of RNase/DNase-free water. Reactions were run in triplicate using a Rotor-Gene Q 5 Plex thermal cycler (Qiagen, Germany) with the following cycling conditions: initial denaturation at 95 °C for 3 min, followed by 40 cycles of denaturation (95 °C, 15 s), annealing (60 °C, 30 s), and extension (72 °C, 30 s).

Relative gene expression was calculated using the 2^−ΔΔCt method. ΔCt was determined by subtracting the Ct of the reference gene (*rpoB*) from the Ct of the target gene. Fold change was then calculated as 2^−ΔΔCt, where ΔΔCt = ΔCt (treated sample) – ΔCt (control). All experiments were performed in three independent biological replicates, each analyzed with four technical replicates. Primer sequences and reagent details are provided in Tables 1 and 2 [14].

**Table 1:** Primers sequence utilized in this study

Primer name	Sequence
*rpoB* (housekeeping gene)	Forward: GGCGAAATGGCWGAGAACCA Reverse: GAGTCTTCGAAGTTGTAACC
*fimH* gene	Forward: TGCAGAACGGATAAGCCGTGG Reverse: GCAGTCACCTGCCCTCCGGTA
*mrkD* gene	Forward: CTGACGCTTTTTATTGGCTTAATGGCGC Reverse: GCAGAATTTCCGGTCTTTTCGTTTAGTAG

**Table 2:** Volumes and concentrations of qPCR reaction mix

Components	Volume (µl)
cDNA template	3
2x maxima SYBR Green qPCR Master Mix	12.5
Forward primer	1
Reverse primer	1
Water, nuclease free	7.5
Total	25

#### Molecular docking study

Molecular docking was performed to investigate the binding interactions between α-amylase and the fimH (PDB: 4XO8) and mrkD (PDB: 3U4K) adhesin proteins as well as MrkH Protein (PDB: 5KEC), a transcription regulator. The 3D structures of the proteins were retrieved from the Protein Data Bank (PDB).

The analysis was performed using MOE-Dock software (version 2020.09). The 2D structure of the α-amylase pigment was illustrated using ChemDraw. Before docking, all non-essential residues were removed, and polar hydrogens were added to the protein chains using MOE’s Protonate 3D protocol with default settings, ensuring proper protonation states for molecular docking simulations.

Partial charges were automatically calculated after energy minimization using the MMFF94x force field in MOE, with convergence set at an RMSD gradient of 0.05 kcal mol⁻¹ Å⁻¹. Ligand preparation involved generating 3D conformations, assigning hydrogens and partial charges, and conducting a systematic search to explore all possible dihedral angle combinations for each ligand [15]. Docking simulations employed the London-dG scoring function and the Triangle Matcher placement method. For each ligand, up to 30 conformers (poses) were generated, with the top five refined poses retained for further analysis. The best ligand conformation was selected based on lowest binding free energy, interaction distance, RMSD (≤ 2 Å), and key amino acid interactions within the binding site.

### Statistical analysis

All experiments were conducted in triplicate to ensure accuracy and reproducibility. Statistical analyses were performed using GraphPad Prism (version 5). One-way analysis of variance (ANOVA) followed by Tukey’s post hoc test was used to compare group means. Differences were considered statistically significant at *p* < 0.05.

## Results

### Biochemical identification of test clinical isolates of *K. pneumoniae*

VITEK^®^2 system 7.01 software correctly identified 25 *K. pneumoniae* isolates, including the control strain (ATCC 700603), giving an accuracy of 99%. The average probability of the interval) = 95%; SD (standard deviation) = 3.98%; variance = 0.16%). Among the 25 isolates identified as *K. pneumoniae*, one isolate had a probability value below the deviation range. The first column shows the cassette well number, (+) for positive results and (-) for negative results as presented in Table 3.

**Table 3:** Biochemical identification of test *K. pneumoniae* clinical isolates using Vitek 2 compact systems, (-) for negative results and (+) for positive results

2	APPA	−	3	ADO	+	4	PyrA	+ 2	5	IARL	−3	7	dCEL	+ 4	9	BGAL	+ 5
10	H2S	−	II	BNAG	.	12	AGLTp	−	13	dGLU	+	14	GGT	+	15	OFF	+
17	BGLU	+	18	dMAL	+	19	dMAN	+	20	dMNE	+	21	BXYL	+	22	BAlap	−
23	ProA	−	26	LIP	.	27	PLE	+	29	TyrA	+	31	URE	+	32	dSOR	+
33	SAC	+	34	dTAG	−	35	dTRE	+	36	CIT	+	37	MNT	+	39	5KG	−
40	ILATK	+	41	AGLU	−	42	SUCT	+	43	NAGA	−	44	AGAL	+	45	PHOS	+
46	GlyA	−	47	ODC	−	48	LDC	+	53	IHISa	.	56	CMT	−	57	BGUR	−
58	0129R	+	59	GGAA	−	61	IMLTa	−	62	ELLM	−	64	ILATa	−			

### MIC and MBC of α-amylase against clinical isolates of *K. pneumoniae*

MIC and MBC results of *B. cereus*-derived α-amylase against twenty-five isolates of *K. pneumoniae* were shown in Table 4. Data of *B. cereus*-derived α-amylase was presented as two groups; group A, which involved 20 test isolates (numbered 1–20) and displayed MIC and MBC of 32 and 64 µg/mL, respectively; nevertheless, group B consisted of 5 bacterial isolates (numbered 21–25) showed MIC and MBC values of 64 µg/mL and 128/mL, respectively. Commercial authentic α-amylase enzyme originated from *B. amyloliquefaciens* was utilized to evaluate and compare the results (MIC = 128 µg/mL, MBC = 256 µg/mL).

**Table 4:** Results of MIC and MBC in µg/ml of *B. cereus*-derived α-amylase on clinical isolates of *K. pneumoniae*

Test	K. pneumoniae isolates group A samples^a^	K. pneumoniae isolates group B samples^b^
**MIC**	32	64
**MBC**	64	128

^a^Twenty isolates numbered from 1 to 20

^b^Five isolates numbered from 21 to 25

### Qualitative assessment of *K. pneumoniae* biofilm formation

**Fig. 1:**
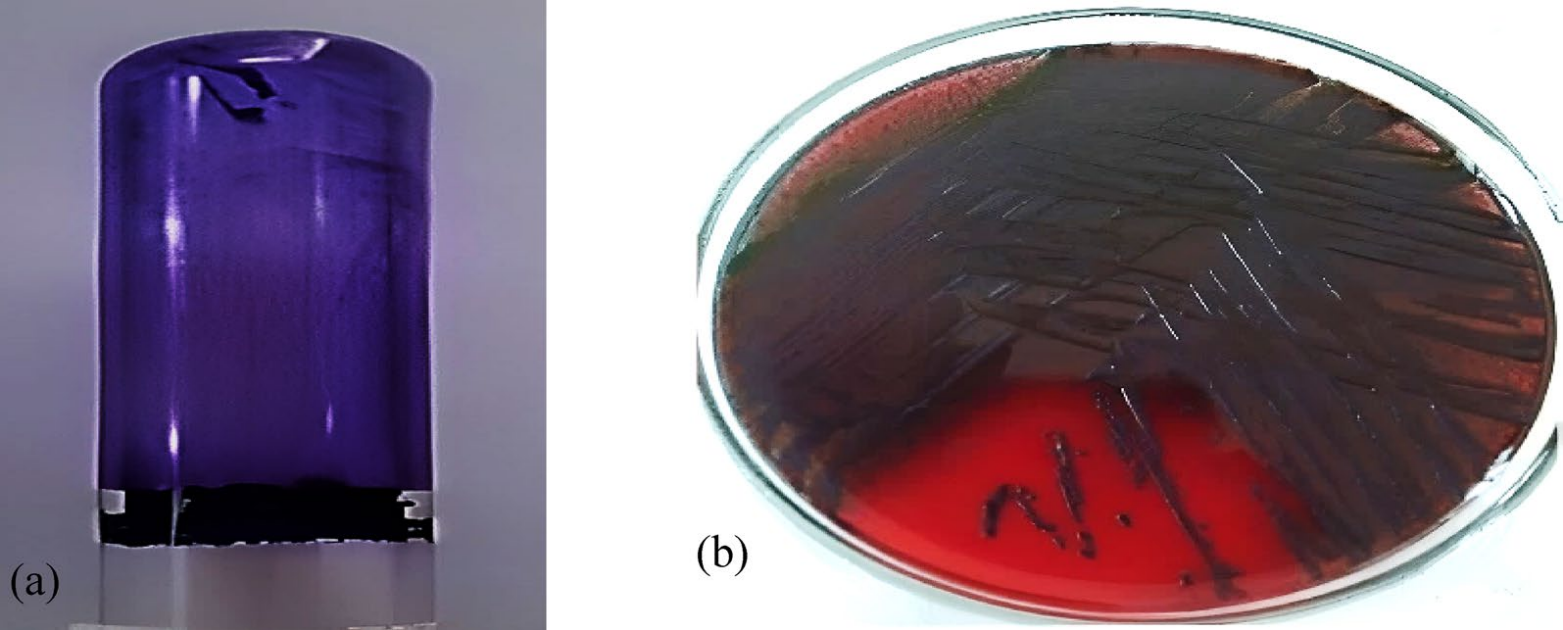
Qualitative detection of biofilm-forming pathogens. (**a**) Visible CV-stained biofilm ring on polystyrene surface showing positive biofilm ability of *K. pneumoniae*. (**b**) black colonies of *K. pneumoniae* on CRA media indicating positive biofilm formation

Screening for biofilm-forming *K. pneumoniae* isolates was done using both the tube as well as Congo red agar methods. As shown in Fig. 1a, visible thick film was observed on the internal side of the tube and the bottom of the tube, indicating biofilm production. A total of 21 (84%) out of 25 isolates gave positive results in both the tube as well as Congo red methods. Additionally, bacterial growth on Congo red agar revealed black colored colonies, indicating biofilm production by the test pathogens (Fig. 1b).

### MBIC and MBEC of commercial and *B. cereus*-derived α-amylase on biofilm-producing *K. pneumoniae* isolates by microtiter plate spectrophotometric assay

As shown in Table 5, MBIC of *B. cereus* α-amylase and commercial *B. amyloliquefaciens* α-amylase on biofilm-forming *K. pneumoniae* isolates was 64 µg/ml and 256 µg/ml, respectively, however, MBEC of these enzymes on pre-formed biofilm was 128 and 512 µg/ml, respectively.

**Table 5:** MBIC and MBEC in µg/ml of ***B. cereus***-derived and commercial amylases against ***K. pneumoniae*** test isolates.

Test	B. cereus-derived α-amylase	Commercial B. amyloliqufaciens alpha amylase
**MBIC**	64	256
**MBEC**	128	512

### Antibiofilm effect of *B. cereus*-derived and commercial α-amylase enzymes against *K. pneumoniae* biofilm by crystal Violet spectrophotometric assay

**Fig. 2:**
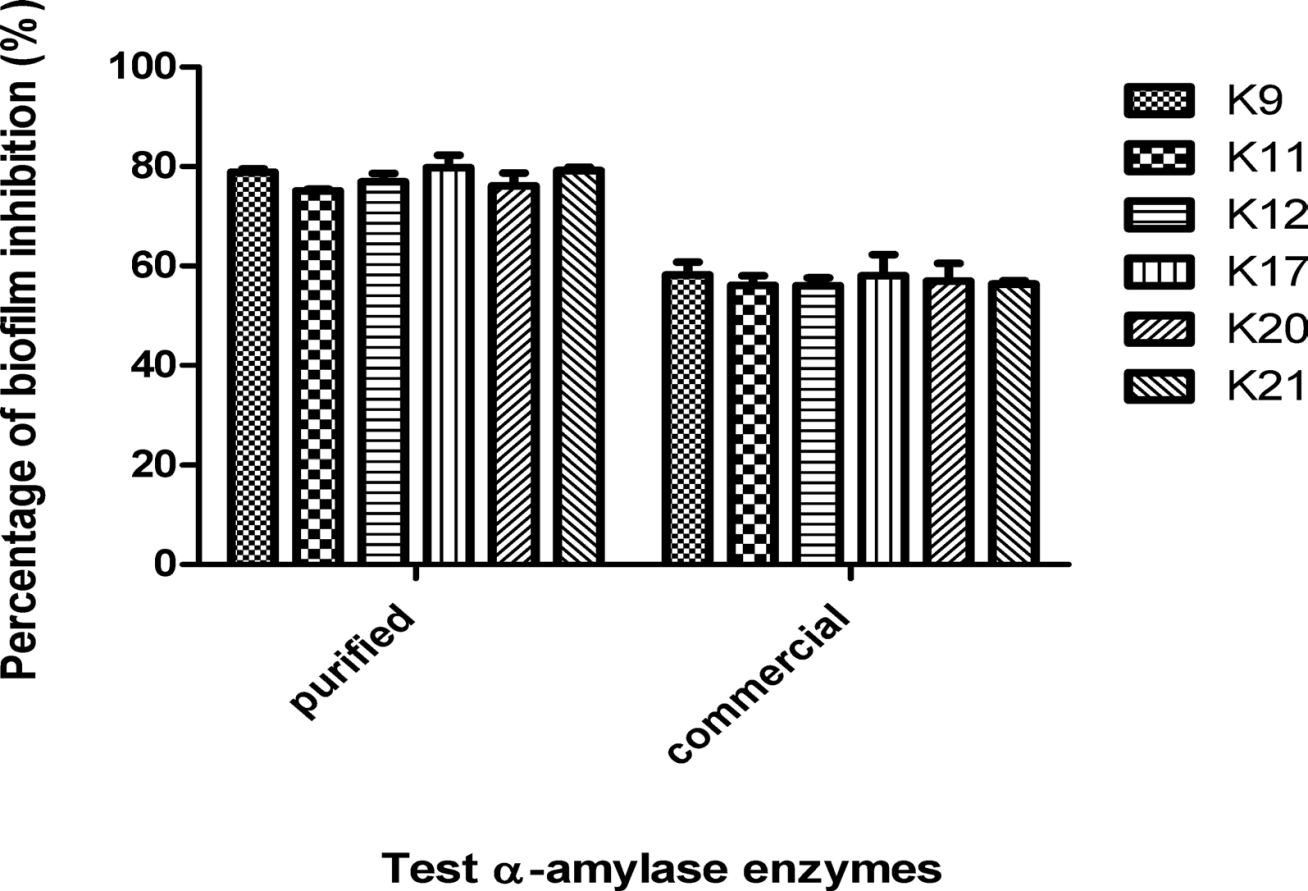
Percentage of biofilm inhibition of *K. pneumoniae* isolates (K9, K11, K12, K17, K20, and K21) after treatment with either of commercial *B. amyloliquefaciens* amylase or *B. cereus*-derived α-amylase. *P* value < 0.001.

Results shown in Fig. 2 present the percentage reduction in biofilm formation exerted by ½ MIC of *B. cereus*-derived or commercial α-amylases against six selected biofilm forming test isolates of *K. pneumoniae*. For the *B. cereus*-derived α-amylase, biofilm was significantly inhibited by ~ 75–79% while commercial α-amylase exerted an inhibitory effect by about ~ 56–58%.

### CLSM results before and after treatment of *K. pneumoniae* with commercial and *B. cereus*-derived α-amylase enzyme, and image J analysis of live/dead cells percentage of this assay

**Fig. 3:**
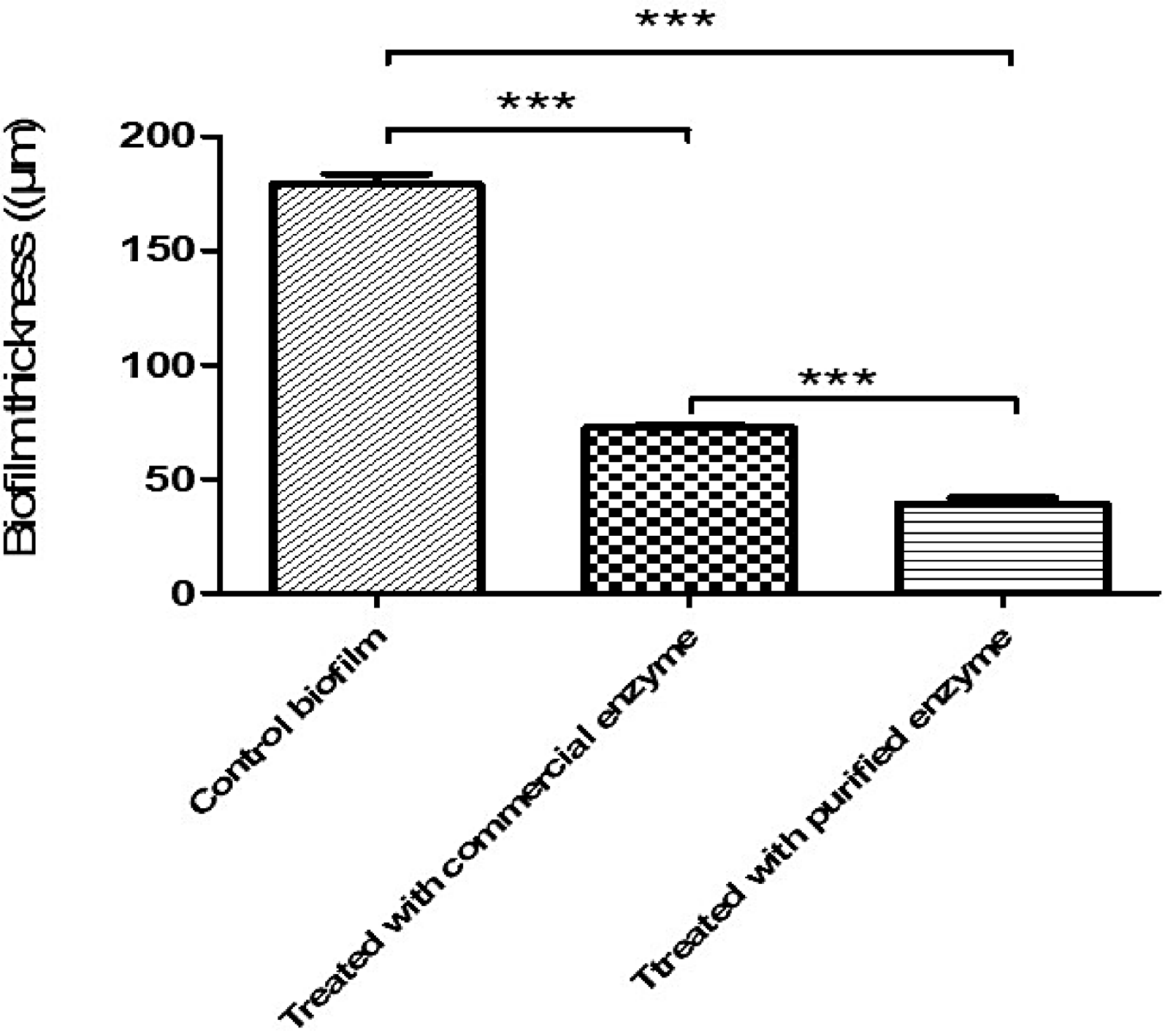
*K. pneumoniae* biofilm thickness before and after treatment with commercial and *B. cereus*-derived α-amylase. For significant differences; *p* < 0.0001.

**Fig. 4:**
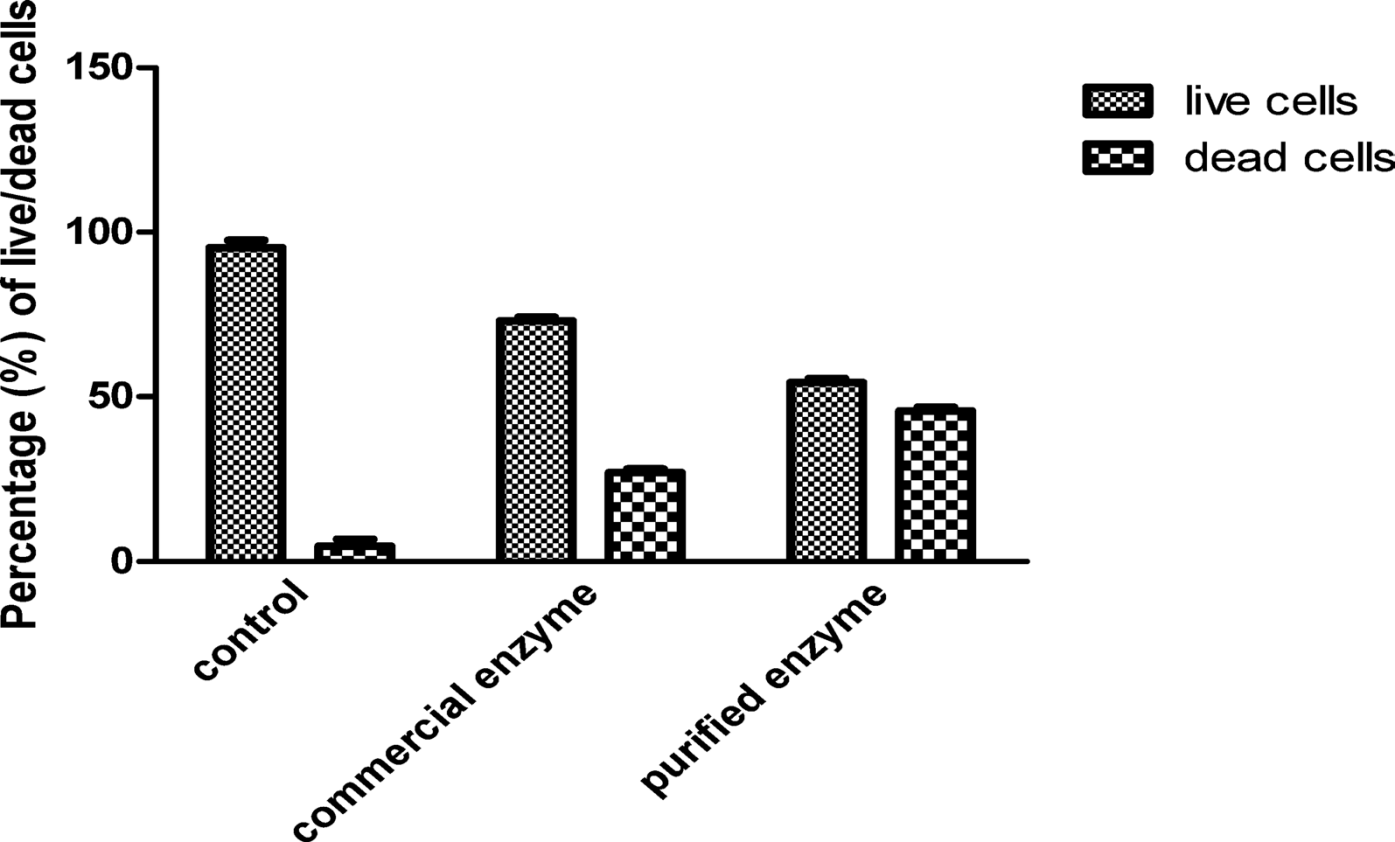
Percentages of live/dead cells of *K. pneumoniae* biofilms before and after treatment using commercial as well as *B. cereus*-derived α-amylase. *P* < 0.0001

**Fig. 5:**
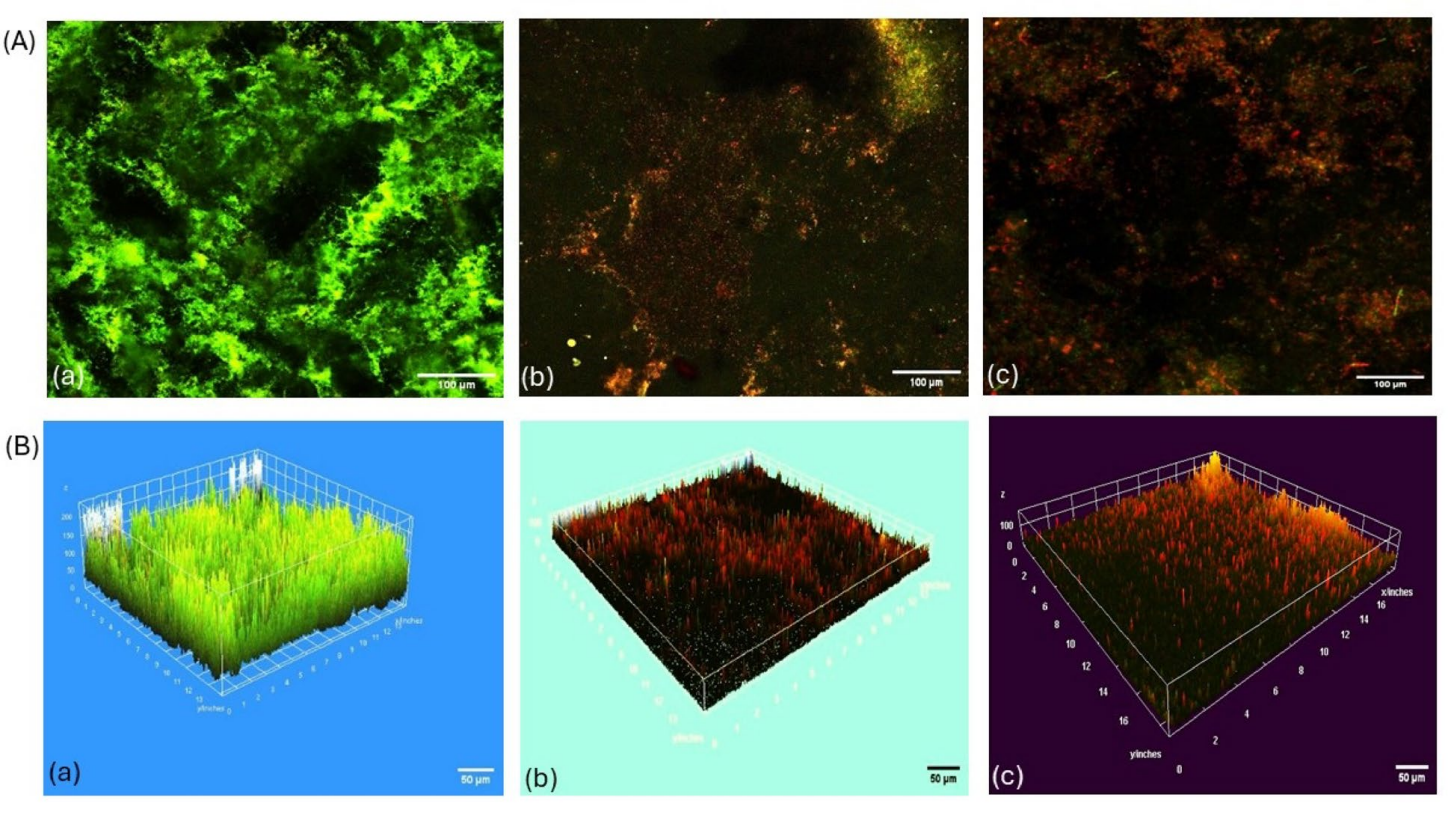
Confocal microscopy (**A**) and 3D-reconstructed (**B**) images of *K. pneumoniae* biofilm stained with acridine orange/propidium iodide (AO/PI). Panel A(a) shows the Z-stack confocal image of an untreated biofilm (control), while A(b) and A(c) display biofilms treated with B. *cereus*-derived α-amylase and commercial α-amylase, respectively. Similarly, panel B(a) presents the 3D reconstruction of the control biofilm, whereas B(b) and B(c) illustrate the effects of *B. cereus*-derived α-amylase and commercial α-amylase treatments on biofilm architecture

**Fig. 6:**
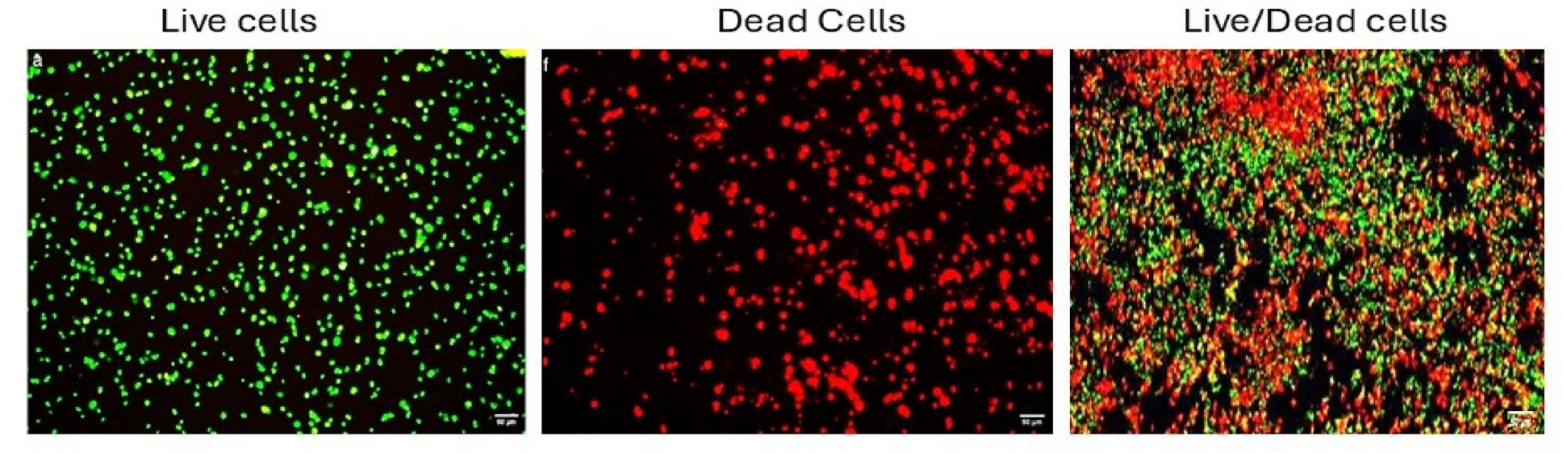
Live/dead cells images generated by image J software displaying ROI of live cells (appeared as green particles) and dead cells (appeared as red particles)

Assessment of biofilm thickness was performed before and after treatment with α-amylases. It was found that the thickness was dropped from 179 μm to ~ 73 and 39 μm following treatment with commercial and *B. cereus*-derived α-amylase enzymes, respectively, as shown in Fig. 3. Additionally, the viability of the microbial cells was assessed in the absence or presence of the enzymes, as shown in Fig. 4. Percentages of live/dead cells of *K. pneumoniae* biofilms were significantly changed following treatment with the *B. cereus*-derived and commercial amylases from 97/3% to ~ 54/46%, and 73/27%, respectively as shown in Fig. 4. Images of CLSM for both the viability of *K. pneumoniae* cells within the biofilm as well as the thickness, were presented in Fig. 5. A representative analysis of pictures by Image J software was presented in Fig. 6.

### Effect of the *B. cereus*-derived α-amylase on *K. pneumoniae* biofilm encoding genes responsible for quorum sensing mechanism

**Fig. 7:**
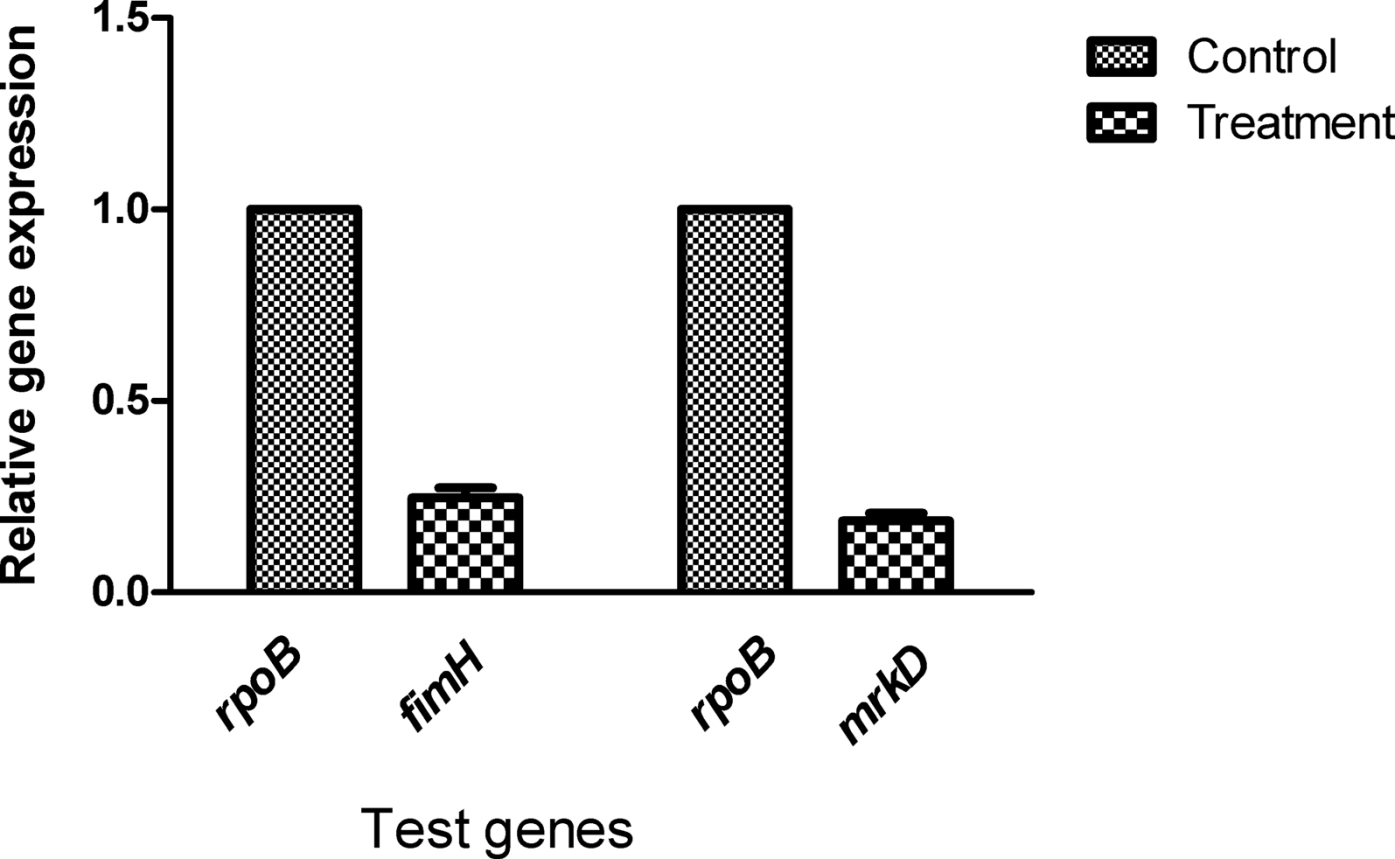
Down expression of quorum sensing genes of *K. pneumoniae* after treatment with ½ MIC of *B. cereus*-derived α-amylase enzyme. *P* < 0.001

As shown in Fig. 7, *fimH* gene expression was dropped to 0.247 ± 0.045 meaning that it was downregulated by 75.3% after treatment of biofilm-producing *K. pneumonia* with ½ MIC (32 µg/ml) of *B. cereus*-derived α-amylase enzyme and *mrkD* gene expression was reduced to 0.187 ± 0.035 denoting that it was downregulated by 81.3% after treatment with the test enzyme.

### Molecular docking

#### FimH adhesin protein (PDB code: 4XO8)

**Fig. 8:**
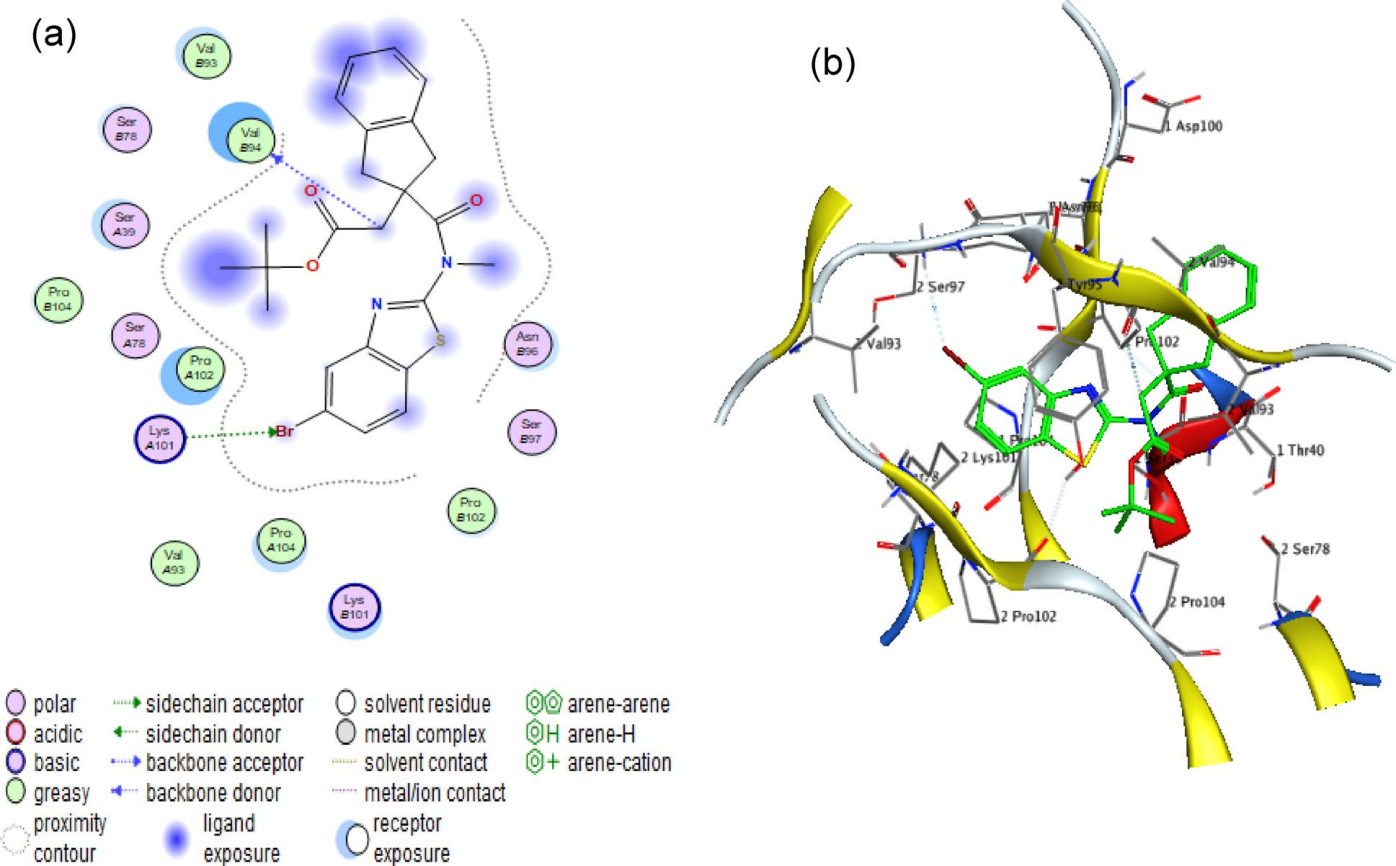
Molecular docking analysis of α-amylase with FimH protein. (**a**) 2D interaction diagram and (**b**) 3D binding pose reveal a stable complex (binding energy: −6.6686 kcal/mol), stabilized by hydrogen bonds with Val94 and Lys101. These interactions suggest potential competitive inhibition of FimH-mediated bacterial adhesion

The molecular docking results demonstrate a strong interaction between α-amylase and the *fimH* adhesin protein, with a binding score of −6.6686 kcal/mol. The 2D and 3D representations illustrate specific hydrogen bonding interactions with key amino acids VAL 94 and LYS 101 as presented in Fig. 8. These precise molecular interactions suggest potential inhibitory effects on *fimH*-mediated bacterial adhesion, warranting further experimental investigation.

#### mrkD - Fimbria adhesin protein (PDB: 3U4K)

**Fig. 9:**
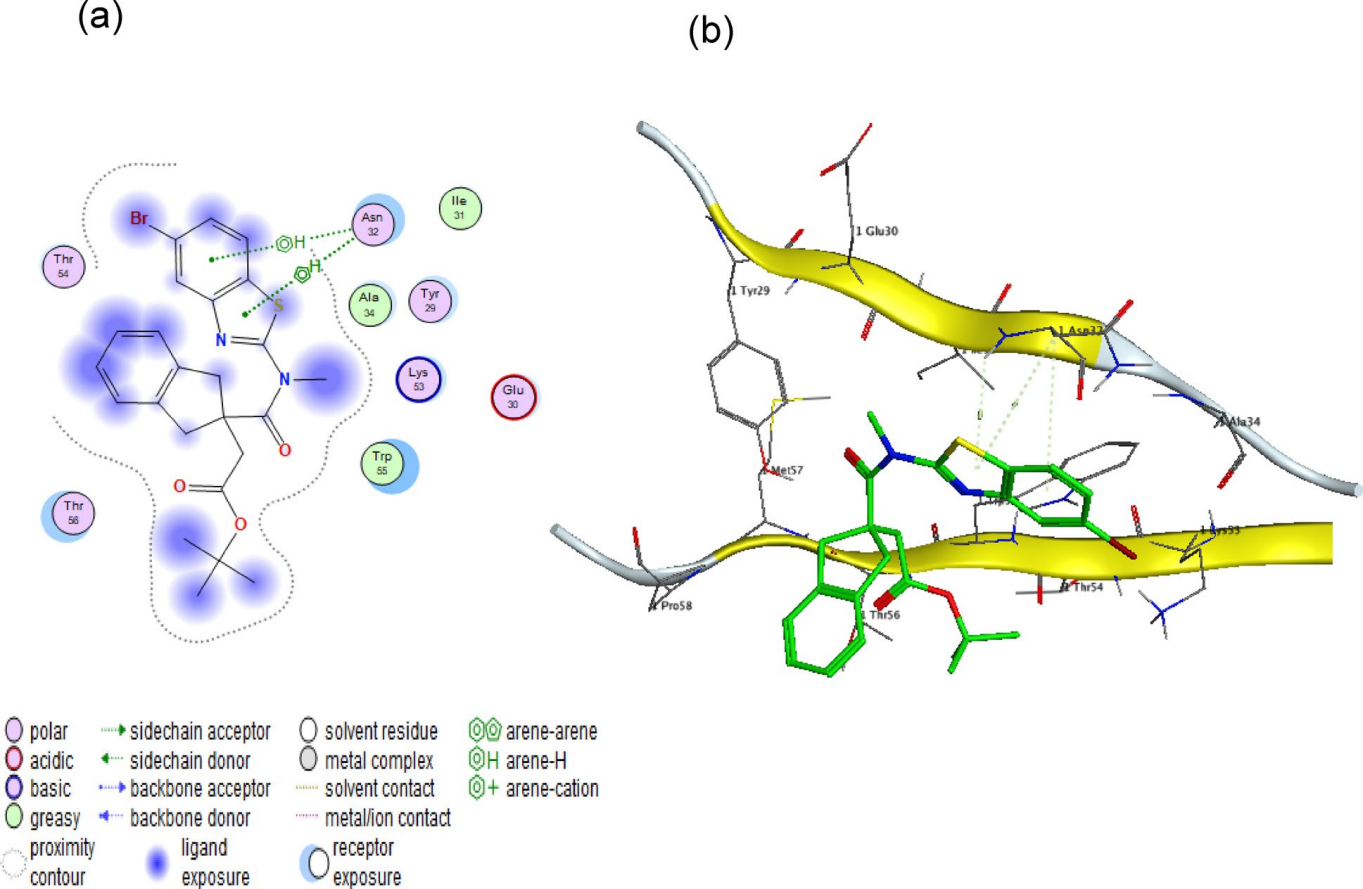
Molecular interaction analysis of α-amylase with mrk-D protein. (**a**) 2D interaction diagram and (**b**) 3D binding pose demonstrate a stable complex (binding energy: −5.8545 kcal/mol), with the fimbria adhesin protein via multiple hydrophobic bonding interactions centered at ASN32 amino acid

The docking analysis reveals significant binding between α-amylase and the *mrkD* fimbrial adhesin, with a binding affinity of −5.8545 kcal/mol. The 2D and 3D visualizations highlight hydrophobic interactions centered around the ASN32 residue as shown in Fig. 9. This distinct binding mode indicates a possible mechanism for disrupting mrkD-dependent bacterial adhesion, which should be validated through subsequent biochemical studies.

#### MrkH protein (PDB: 5KEC)

**Fig. 10:**
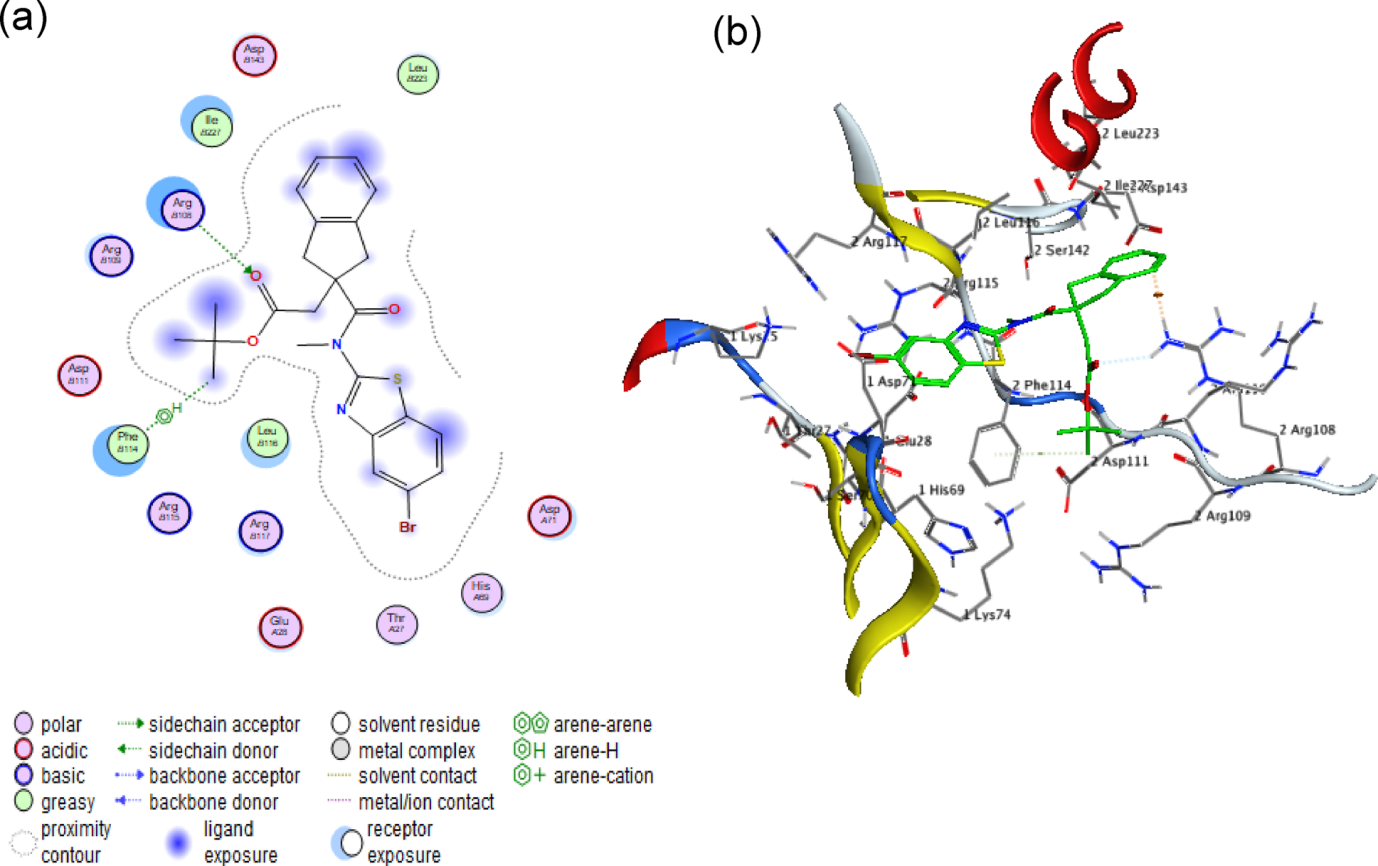
Molecular docking analysis of the MrkH–α-amylase interaction. (**a**) 2D ligand interaction diagram and (**b**) 3D binding pose reveal a stable complex (binding energy: −6.7735 kcal/mol), stabilized by a hydrogen bond with Arg108 and hydrophobic interactions with Phe114

The molecular docking analysis revealed a stable binding interaction between the MrkH protein and α-amylase, with a favorable binding score of −6.7735 kcal/mol. The complex formation was primarily stabilized by a hydrogen bond with ARG108, contributing to polar interactions, as well as hydrophobic interactions involving PHE114, enhancing binding affinity (Fig. 10).

## Discussion

Antibiotic resistance in *K. pneumoniae* represents a formidable global health challenge, primarily driven by the pathogen’s ability to acquire and disseminate resistance genes through horizontal gene transfer. The emergence of multidrug-resistant (MDR) and extensively drug-resistant (XDR) strains has severely limited therapeutic options, posing significant threats in both hospital and community settings [16]. A critical factor contributing to the resilience of *K. pneumoniae* is its capacity to form biofilms—structured microbial communities encased in a self-produced extracellular polymeric matrix. Biofilms serve as a protective barrier, reducing antibiotic penetration and fostering a localized microenvironment that promotes the exchange of resistance determinants [17]. Furthermore, the biofilm mode of growth enhances the expression of stress-response genes, thereby increasing bacterial survival under antimicrobial pressure. Clinical studies have demonstrated biofilm-associated infections caused by *K. pneumoniae* exhibit higher treatment failure rates than planktonic infections [17–19]. This dual threat of antibiotic resistance and biofilm formation underscores the urgent need for novel therapeutic strategies targeting resistance mechanisms and biofilm integrity.

In the current study, the minimum inhibitory concentration (MIC) and minimum bactericidal concentration (MBC) values obtained for the *B. cereus*-derived α-amylase demonstrated substantial bactericidal activity against *K. pneumoniae* isolates. For Group A isolates, the MIC and MBC values were 32 µg/mL and 64 µg/mL, respectively, while for Group B isolates, these values increased to 64 µg/mL and 128 µg/mL. Notably, these results suggest variability in susceptibility among the clinical isolates, potentially attributable to differences in their genetic or phenotypic characteristics. In comparison, the commercial α-amylase from *Bacillus amyloliquefaciens* exhibited comparatively higher MIC and MBC values, highlighting the superior antimicrobial efficacy of the *B. cereus*-derived α-amylase. This is consistent with prior studies demonstrating that microbial enzymes can exhibit strain-specific activity due to variations in enzyme-substrate interactions [20].

The ability of the *B. cereus*-derived α-amylase to inhibit biofilm formation and disrupt pre-formed biofilms of *K. pneumoniae* was evident in the microtiter plate and CLSM analyses. The MBIC and MBEC values for the *B. cereus*-derived α-amylase enzyme (64 µg/mL and 128 µg/mL, respectively) were significantly lower than those for the commercial enzyme. These findings were consistent with its potent biofilm-disrupting ability, as evidenced by 75–79% reduction in biofilm formation by ½ MIC of *B. cereus*-derived α-amylase compared to 56–58% by the commercial enzyme. This might be due to the distinct behaviors and dynamics of planktonic versus sessile cells, in addition to the effect of α-amylase as a matrix-degrading enzyme [21]. Furthermore, a marked decrease in biofilm thickness was recorded by *B. cereus*-derived α-amylase (from 179 μm to ~ 39 μm; about 78% reduction). Literature demonstrated that α-amylase from *Bacillus subtilis* affected the biofilm structures (44–62%) of different clinical isolates, including; MRSA, *Vibrio cholerae*, and *Pseudomonas aeruginosa*, influencing the biofilm growth process [22]. Moreover, it has been observed that the biofilm architecture of *Pseudomonas aeruginosa* (82%) can be compromised by enzymes such as protease (10 U/mg), amylase (8 U/mg), and pectinase (10.086 U/mg), which are generated via solid-state fermentation by the fungal species *Aspergillus clavatus* [23]. The α-amylase derived from *Penicillium janthinellum* exhibited more than 80% antibiofilm efficiency against *Salmonella enterica*, *P. aeruginosa*, *Staphylococcus aureus*, and *Escherichia coli* [24]. However, Ramakrishnan and co-workers reported that bovine α-amylase demonstrated very poor activity to degrade polysaccharides of biofilm-forming *K. pneumoniae* isolates (∼25% reduction) [24]. The decrease in biofilm biomass and thickness demonstrated in our findings probably underscores the ability of our *B. cereus*-derived α-amylase enzyme to target extracellular polymeric substances (EPS), which form the structural backbone of biofilms, via a degradation process that diminishes the carbohydrates that constitute the EPS framework. Previous studies have highlighted the importance of disrupting EPS to achieve effective biofilm eradication [22, 25].

Numerous earlier studies have investigated how natural compounds, like acids and essential oils, influence quorum sensing genes in *K. pneumoniae*. Research focusing on the impact of the bacterial enzyme α-amylase on the expression of biofilms in Gram-negative bacteria is quite scarce. A limited study related to this phenomenon indicated that the QS mechanism in Gram-negative bacteria such as *K. pneumoniae* involves specific molecules called acylhomoserine lactones (AHLs). This molecule has a lactone ring that plays a significant role in QS signaling and the formation of biofilms; certain extracellular enzymes like lipases, proteases, and amylases can break this lactone ring in pathogenic bacterial cells, disrupting the expression of quorum sensing molecules and their coding genes. In the present study, the downregulation of fimbrial adhesin genes (*fimH* and *mrkD*) following treatment with the *B. cereus-derived* α-amylase underlines its anti-QS potential. Gene expression analysis revealed a significant reduction in *fimH* and *mrkD* expression by 75.3% and 81.3%, respectively. These genes are critical for adhesion and biofilm formation in *K. pneumoniae* and are regulated by quorum sensing mechanisms [25, 26]. The observed downregulation suggests that the *B. cereus* derived α-amylase enzyme interferes with QS signaling pathways, thereby inhibiting biofilm formation and maintenance. This is in agreement with the findings of Abo-Kamer et al. [25], who reported similar anti-QS activities of α-amylase from *B. subtilis* against uropathogenic *E. coli*.

While both the *B. cereus* derived α-amylase and commercial ones exhibited antimicrobial and antibiofilm activities, the *B. cereus* derived α-amylase consistently outperformed its commercial counterpart. This enhanced efficacy could be attributed to differences in enzyme purity, structure, or activity. The superior performance of the *B. cereus* derived α-amylase enzyme in reducing biofilm viability (live/dead ratio: ~54/46%) and thickness further highlights its therapeutic potential. Such findings underscore the importance of enzyme source and purification in determining efficacy. The ability of the *B. cereus* derived α-amylase to target both planktonic cells and biofilms has significant implications for treating biofilm-associated infections, particularly in the context of multidrug resistance. The enzyme’s dual action in disrupting biofilms and inhibiting QS provides a multifaceted approach to infection control, potentially reducing the likelihood of resistance development. Our findings highlight the potential of α-amylase as a promising therapeutic adjunct to combat biofilm-associated infections caused by *K. pneumoniae*, a pathogen responsible for life-threatening conditions such as pneumonia, bloodstream infections, and urinary tract infections [25, 26]. The enzyme’s dual functionality, combining enzymatic and antimicrobial properties, underscores its utility in addressing the global challenge of antimicrobial resistance.

The antibiofilm activity of α-amylase was confirmed by the docking results that reveal that α-amylase forms stable complexes with both fimH and mrkD adhesins, mediated by distinct interactions. The hydrogen bonds with VAL 94 and LYS 101 in fimH and the hydrophobic interactions with ASN32 in mrkD underscore the selectivity of these bindings. The higher binding score for fimH suggests a stronger affinity, which may correlate with more effective inhibition of bacterial adhesion. These computational insights provide a foundation for future studies aimed at developing α-amylase-based therapeutic strategies targeting bacterial adhesins. Similar interactions between bacterial adhesins and inhibitory proteins have been reported in prior studies [27, 28]. MrkH acts as a transcription activator for the *mrk* gene cluster, controlling the expression of *mrkHI* and possessing a PilZ domain. It binds to the upstream region of the *mrkA* promoter, stimulating transcription of the *mrkABCDF* operon. As a result, MrkH plays a key role in triggering the expression of genes responsible for type 3 fimbriae production [29–31]. Furthermore, the interaction between α-amylase and mrkH protein, known as the “biofilm switch”, was also studied by molecular docking. Data showed that a stable interaction between α-amylase and the MrkH regulatory protein (−6.7735 kcal/mol), mediated by a hydrogen bond (Arg108) and hydrophobic contacts (Phe114). This interaction suggests that α-amylase might modulate MrkH’s activity, potentially interfering with its ability to trigger expression of type 3 fimbriae genes and hence, biofilm formation.

In conclusion, this study highlights the potent anti-biofilm and antibacterial activities of a novel α-amylase enzyme from *Bacillus cereus* against multidrug-resistant *K. pneumoniae* clinical isolates. The enzyme demonstrated significant biofilm inhibition and eradication capabilities, as evidenced by a 79% reduction in biofilm thickness and a favorable live/dead cell ratio of ~ 54/46%). The MIC and MBC values, coupled with the substantial downregulation of key biofilm-associated genes (*fimH* and *mrkD*), underscore its quorum-quenching and bactericidal effects. These findings suggest that α-amylase could serve as a promising candidate for combating biofilm-associated infections caused by *K. pneumoniae*.

Further research is warranted to elucidate the precise mechanisms underlying the enzyme’s anti-QS and antibiofilm activities. Additionally, in vivo studies are needed to validate its efficacy and safety. Investigating the synergistic effects of α-amylase with conventional antibiotics could also provide insights into combination therapies for MDR bacterial infections.

## Data Availability

All data generated or analysed during this study are included in this published article.
